# Oral Bioavailability of Monoclonal Antibody

**DOI:** 10.3390/pharmaceutics18010022

**Published:** 2025-12-23

**Authors:** Ashwni Verma, Shengjia Wu, Dhaval K. Shah

**Affiliations:** Department of Pharmaceutical Sciences, School of Pharmacy and Pharmaceutical Sciences, The State University of New York at Buffalo, Buffalo, NY 14214, USA; ashwnive@buffalo.edu (A.V.); swu47@buffalo.edu (S.W.)

**Keywords:** antibody, pharmacokinetics (PK), oral bioavailability

## Abstract

**Background/Objectives:** Despite significant interest in the oral delivery of antibodies, the oral bioavailability of monoclonal antibodies (mAbs) is not known to date. To find out an answer to this question, we have performed a preclinical investigation in mice and rats, using a non-binding humanized mAb trastuzumab as the prototype molecule. **Methods:** The antibody was administered at the dose of 100 mg/kg in mice and rats, and plasma pharmacokinetics (PK) was measured for 14 days. Published plasma PK of trastuzumab in mice and rats obtained after intravenous administration was also used for the analysis. Non-compartmental analysis (NCA), as well as compartmental modeling of PK data, was performed to estimate the oral bioavailability of the antibody in mice and rats. **Results:** It was found that the oral bioavailability of mAb in rats and mice determined using NCA was 0.027% and 0.014%, respectively. The model-estimated oral bioavailability of the mAb in rats and mice was 0.025% and 0.013%, respectively. The rate of absorption of the mAb from the gastrointestinal tract was found to be the same between rats and mice, as 0.78 h^−1^. **Conclusions:** Overall, the oral bioavailability of the humanized mAb in rodents was found to be around 0.02%, suggesting only 1 out of 5000 mAb molecules administered orally makes it to the systemic circulation. To the best of our knowledge, this is the first study to report an absolute oral bioavailability value for a mAb. It remains to be seen if the observed value of 0.02% is generalizable across mAb molecules and other animal species, including humans.

## 1. Introduction

Oral delivery of protein therapeutics remains a holy grail for pharmaceutical scientists [1]. Despite well-recognized impediments to the oral delivery of proteins, scientists remain undeterred and have made continuous progress in this field for decades [2,3]. With the tremendous therapeutic success of monoclonal antibodies (mAbs), these biologics have become the standard of care for numerous conditions, including malignancies and autoimmune diseases [4,5]. However, currently, mAbs are mainly given intravenously or subcutaneously, which limits patient compliance and accessibility. Consequently, the oral delivery of mAbs has been pursued with great interest. While early efforts focused on providing passive immunization within the gastrointestinal tract [6,7], scientists are now pursuing innovative strategies and state-of-the-art technologies to enable systemic delivery of mAbs following oral administration [8,9,10]. Considering the long half-life of mAbs, the frequency of administration for these molecules can range from weeks to months, which allows one to explore unconventional routes of administration for their delivery [11] while maintaining commercial viability. In fact, several mAbs have already been evaluated in the clinic for therapeutic application following oral administration [12,13,14].

Despite numerous publications evaluating the oral delivery of mAbs in the preclinical and clinical setting, the oral bioavailability of mAb remains unknown to the best of our knowledge. While it is often reported that mAbs have very low oral bioavailability, which is less than 20% [11], less than 5% [6], or less than 1–2% [15], there is no published value for oral bioavailability of mAbs to date. One of the main reasons for this may be the high dose of mAbs that would be needed for oral delivery so that systemic concentrations of mAbs can be detectable by the current analytical methods. Consequently, there are no published reports on systemic pharmacokinetics (PK) of mAbs following oral administration, and the oral bioavailability of mAbs remains unknown. To find out an answer to this important question, in this manuscript, we have performed preclinical investigations in mice and rats using a non-cross reactive humanized mAb and determined the absolute oral bioavailability of antibodies. The quantitative value of the absolute oral bioavailability of antibody discovered here helps assess the feasibility of attaining systemic exposure of antibody following oral delivery and allows one to estimate the oral dose of antibody needed to reach therapeutic exposure at the site-of-action.

## 2. Materials and Methods

### 2.1. Antibody Production, Purification, and Characterization

Trastuzumab was selected as the model mAb for this project due to its strict specificity for human HER2 and lack of cross-reactivity with the rodent homolog [16], ensuring no target binding in mice or rats. Trastuzumab was produced in-house using our CHO cell production system [17]. Antibodies from culture media were purified using the HiTrap^®^ Protein G column (Cytiva, Marlborough, MA, USA). The purity of the antibody was confirmed using gel electrophoresis, and its ability to bind to human HER2 was confirmed using flow cytometry.

### 2.2. Pharmacokinetic Study

All animal procedures were approved by the Institutional Animal Care and Use Committee (IACUC) at the State University of New York at Buffalo. Male mice and male rats were used to perform the oral bioavailability study. Total 10 six-week-old c57bl/6 mice were purchased from Jackson Lab (Bar Harbor, ME, USA), and 10 six-week-old Sprague-Dawley rats were purchased from Taconic Biosciences (Germantown, NY, USA). Around 100–200 µL of antibody solution in PBS, corresponding to 100 mg/kg of the oral dose, was administered using oral feeding gavage to all animals that were fasted for at least 2 h. At each predetermined time point, blood samples were collected from 3 animals via the retro-orbital sinus in mice and the saphenous vein in rats. Plasma was isolated from the blood by centrifuging at 5000 rpm for 10 min and stored at −80 °C until further analysis.

The animal strains, experimental design, and analytical methods used for the oral PK studies were intentionally kept the same as those used in our previously published IV PK study of trastuzumab to ensure full comparability [17,18]. For the IV study, mice and rats were administered with 10 mg/kg of trastuzumab via penile vein injection or tail vein injection, respectively. Blood samples were collected at predetermined time points and processed as mentioned above. Trastuzumab concentrations in mouse and rat plasma were determined using the ELISA detailed below.

### 2.3. Measurement of Antibody Concentrations Using ELISA

Sandwich ELISA was used to determine trastuzumab concentrations in mouse and rat plasma [17,18]. Blank, standard curve, and quality control samples were prepared in the same matrix as plasma samples. Briefly, F(ab)2 goat anti-human IgG-Fc (Bethyl Laboratories, Inc., Montgomery, TX, USA) was used as the capture antibody, plates were blocked using 1% BSA, and alkaline phosphatase-conjugated anti-human IgG-F(ab)2 antibody (Bethyl Laboratories, Inc.) was used as the detection antibody. The plate was developed with PNPP (Thermo Fisher, Waltham, MA, USA) solution in diethanolamine (Thermo Fisher, Waltham, MA, USA), and the change in absorbance was measured at 405 nm for 30 min with Filter Max F-5 microplate analyzer.

### 2.4. Pharmacokinetic Analysis

For comparison with the 100 mg/kg oral dose, plasma concentrations from the previously published 10 mg/kg IV study were dose-normalized by multiplying all concentrations by a factor of 10. This normalization assumes linear PK, as trastuzumab does not bind endogenous targets in rodents and doses in the 10–100 mg/kg range does not saturate FcRn. Noncompartmental analysis of PK data was performed using SimBiology^®^ (MATLAB 2023a). The area under the plasma concentration-time curve (AUC_0-last_) obtained from intravenous and oral PK profiles was used to calculate the oral bioavailability of mAb in each animal species. Oral and intravenous PK profiles of trastuzumab in each species were also fitted simultaneously using a 2-compartment linear PK model to estimate the absolute oral bioavailability of mAb in mice and rats. Maximum Likelihood (ML) estimation method in ADAPT 5 software was used for model fitting.

## 3. Results and Discussion

**Figure 1:**
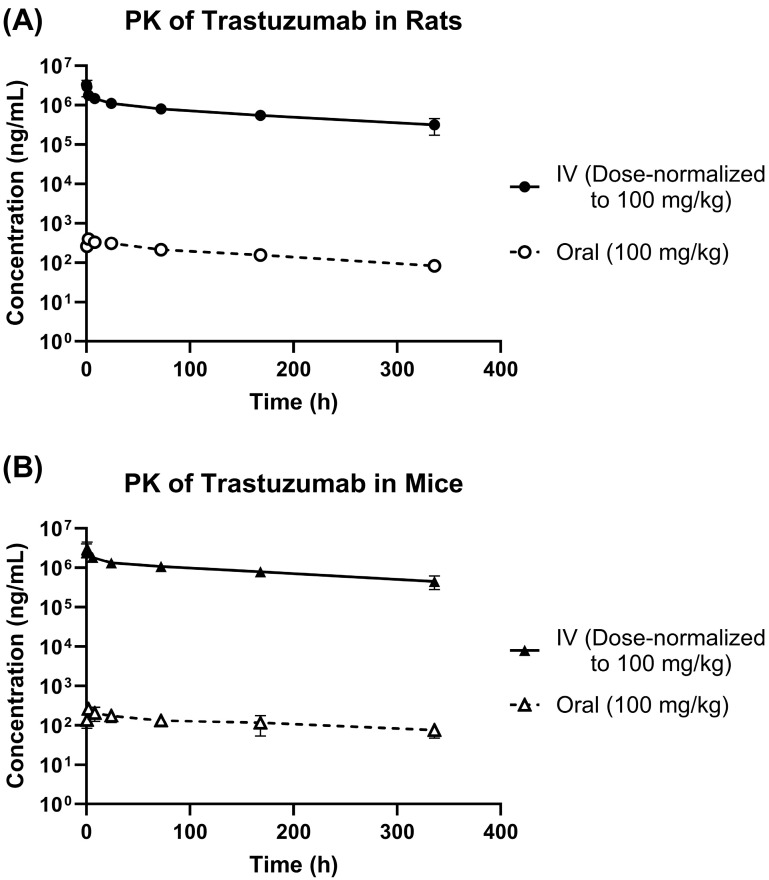
Preclinical PK of trastuzumab. (**A**) Plasma PK of trastuzumab in rats following intravenous (IV) and oral administration of 100 mg/kg dose. (**B**) Plasma PK of trastuzumab in mice following IV and oral administration of 100 mg/kg dose. Note: IV data was dose-normalized to 100 mg/kg for comparison.

**Figure 2:**
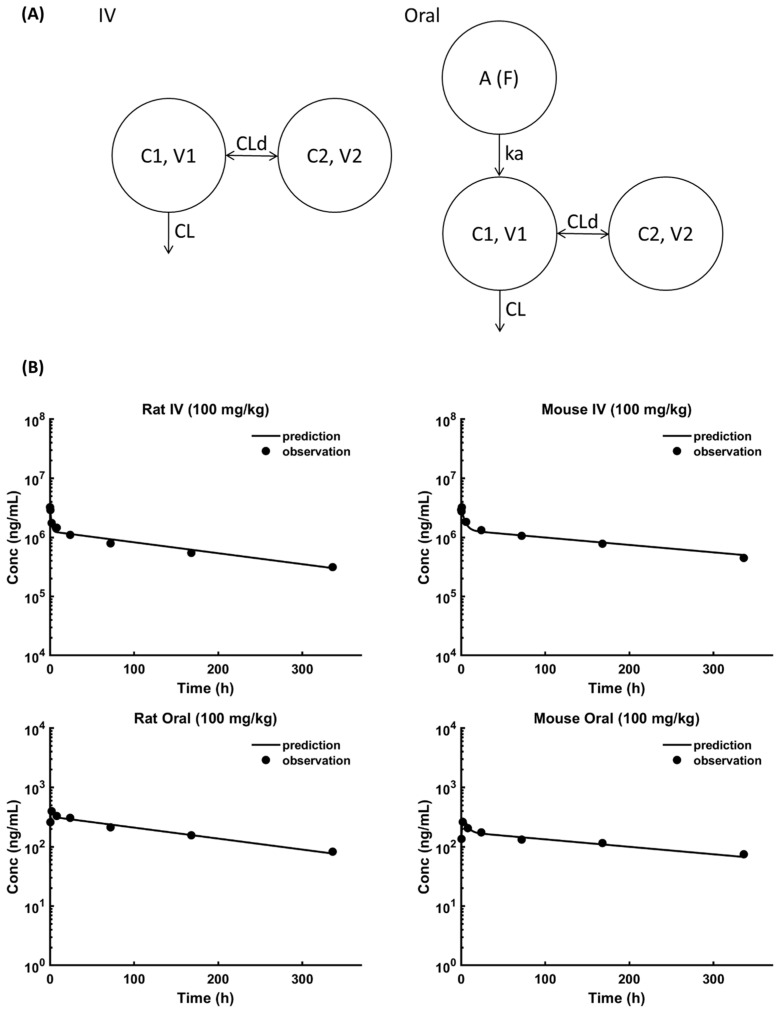
(**A**) Schematic of the two-compartment model that was used to characterize the plasma concentration vs. time profiles of trastuzumab in rats and mice following intravenous and oral administration. (**B**) Model-fitted PK profiles superimposed over the observed data.

Figure 1A,B show plasma PK of trastuzumab in rats and mice, following IV and oral administration of 100 mg/kg dose. Table 1 shows the results from the non-compartmental analysis of these PK profiles. As shown in the table, the oral bioavailability of trastuzumab in rats, calculated using AUC_0-last_ was 0.027%. The oral bioavailability of trastuzumab in mice was found to be 0.014%. The PK data obtained following IV and oral administration were also simultaneously characterized using a 2-compartment PK model with linear clearance (Figure 2A). Figure 2B shows the model fitting results, and Table 2 shows the estimated model parameters. All the parameters were estimated with good precision (Table 2), and the model was able to capture all the PK profiles very well (Figure 2). Model-estimated values for the oral bioavailability of trastuzumab in rats and mice were 0.025% and 0.013%, respectively. These values were very similar to the bioavailability values obtained from the non-compartmental analysis. Interestingly, the model estimated value for the rate of oral absorption of trastuzumab (i.e., ka) was the same, 0.78 h^−1^. This value suggests that the rate of antibody absorption from the gastrointestinal tract of mice and rats is very fast, with an absorption half-life of less than an hour.

The low oral bioavailability of mAbs is mainly due to the extensive proteolytic degradation in the GI tract, as well as low passive permeability across the intestinal epithelium [19]. Proteases such as pepsin and pancreatic enzymes can rapidly cleave antibody molecules, and the large size and high hydrophilicity of intact mAbs further limit efficient epithelial transport. Although FcRn-mediated transcytosis in the intestinal epithelium may facilitate IgG transport and has been shown in some experimental settings to improve protein bioavailability [20,21], the low bioavailability measured here suggests that FcRn binding alone is insufficient to overcome the dominant barriers limiting oral absorption of mAbs.

While the oral bioavailability values of antibodies in mice and rats were slightly different, they were within two-fold of each other and can be considered very similar. On average, the oral bioavailability of antibodies was found to be around 0.02%, which suggests that 1 in 5000 mAb molecules dosed orally makes it through the intestinal barrier and into the systemic circulation. Obviously, such a low bioavailability is commercially nonviable, and supports the ongoing efforts to invent novel technologies to increase the oral bioavailability of mAbs [8,10,22]. However, considering the acceptable bioavailability of mAbs via alternate routes (e.g., 50–100% bioavailability following subcutaneous administration [23]), it is hard to imagine commercial success for oral delivery technologies for mAbs unless they can increase the bioavailability by thousands of folds. Recent device-based oral delivery platforms, such as ingestible capsules that inject large molecules into the stomach wall, have reported high systemic exposures for monoclonal antibodies [8]. However, these systems achieve systemic delivery through trans enteric injection rather than absorption across the gastrointestinal epithelium and therefore represent a fundamentally different delivery mechanism from the natural gastrointestinal (GI) absorption process evaluated in the present study.

Since all the data generated to determine the oral bioavailability of mAb in mice and rats were generated using an antibody with human Fc domain, which has stronger binding to rodent FcRn, it is important to note that the bioavailability of this mAb in humans may be different. In addition, because oral gavage bypasses the mouth and throat, the measured value of ~0.02% may slightly overestimate the true bioavailability achieved through a typical oral route. Nonetheless, the values reported here, which are very low, can be used as the most optimistic value of oral bioavailability for mAbs in humans. Considering this is the first report that determines the absolute oral bioavailability of mAb administered as such, it also remains to be seen if these values are generalizable to other antibodies and other animal species, including humans.

## Figures and Tables

**Table 1:** Results from the non-compartmental analysis of PK profiles shown in Figure 1.

Parameter	Unit	Rat		Mouse	
		IV	Oral	IV	Oral
**Cmax**	ng/mL	3.24 × 10^6^	3.98 × 10^2^	3.23 × 10^6^	2.62 × 10^2^
**AUC_0-t_**	ng×h/mL	2.17 × 10^8^	5.80 × 10^4^	2.93 × 10^8^	4.00 × 10^4^
**CL**	mL/h/kg	0.33		0.23	
**F**	%		0.027		0.014

**Table 2:** Parameter values estimated by fitting 2-compartment PK model to observed concentration vs. time profiles of antibodies in rats and mice obtained following IV and oral administration.

Parameter	Unit	Rat		Mouse	
		Value	CV (%)	Value	CV (%)
**CL**	mL/h/kg	0.33	4.38	0.21	5.67
**CLd**	mL/h/kg	14.06	18.72	4.31	19.19
**V1**	mL/kg	28.86	7.74	31.68	5.21
**V2**	mL/kg	48.79	8.29	41.18	9.16
**F**	%	0.025	4.98	0.013	4.60
**ka**	h^−1^	0.78	14.01	0.78	12.18

## Data Availability

All data generated are provided in the manuscript.
